# Optimization and analysis of rib working resistance in push-the-bit rotary steerable drilling system

**DOI:** 10.1371/journal.pone.0308857

**Published:** 2024-12-16

**Authors:** Jianbo Jia, Youhong Sun, Ke Gao, Yan Zhao, Bing Li, Xu Li

**Affiliations:** 1 School of Engineering and Technology, China University of Geosciences (Beijing), Beijing, China; 2 China Oilfield Services Limited, Beijing, China; 3 College of Construction Engineering, Jilin University, Changchun, Jilin, China; GH Raisoni College of Engineering and Management Pune, INDIA

## Abstract

The rib working resistance of push-the-bit rotary steerable drilling system seriously affects its dynamic performance. For example, the backing pressure problem caused by steps in the well wall during the working process of CNOOC Welleader static push-the-bit rotary steerable drilling tool is discussed in this paper. Based on the structure and working principle of CNOOC Welleader system, a corresponding theoretical model of force analysis is established, and an optimal design method to reduce the backing pressure problem is proposed in combination with numerical simulation and experimental research. The results show that changing the front chamfer area of Welleader system from circular arc to polyline is beneficial to reducing the backing pressure problem. The simulation results show that for steps of 1mm, 2mm and 3mm height, the optimal polyline angle is concentrated in the range of 10°-11°, in which the Angle of 10.5° has a good performance against the steps of three heights. Finally, by making corresponding test blocks, it was found that the peak value of forward resistance when the 10.5°test block crossed the steps of 1mm, 2mm and 3mm height decreased by 19.8%, 25.0% and 13.9% respectively, and the mean value decreased by 30.8%, 27.2% and 24.1% respectively, which was close to the simulation results, and verified the accuracy of the finite element analysis results.

## Introduction

**Fig 1:**
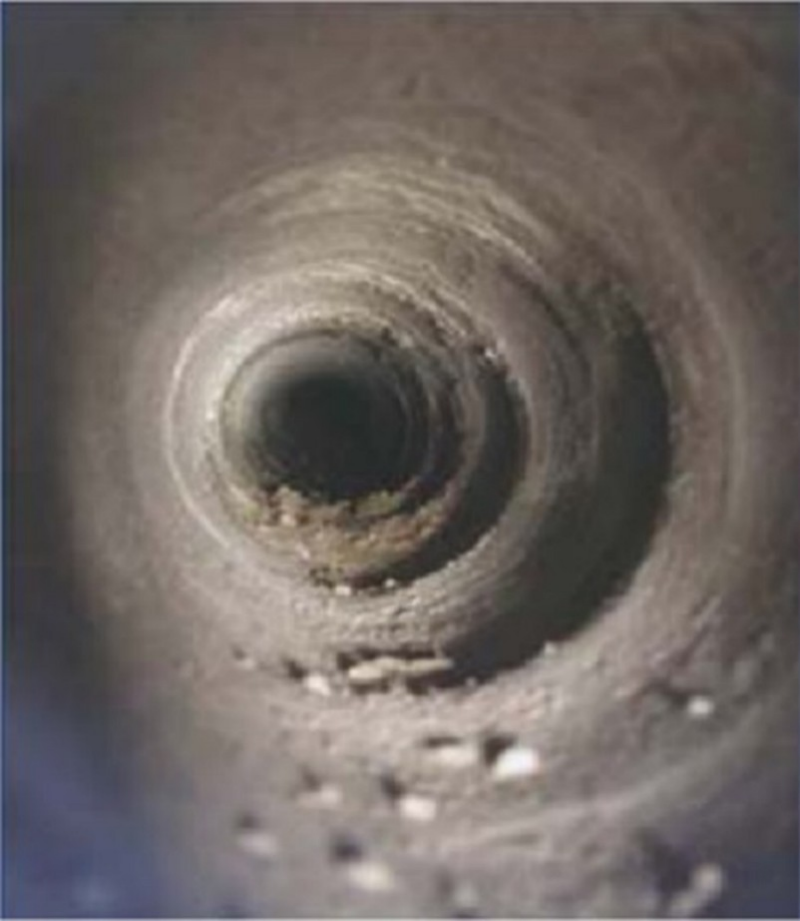
Borehole quality of push-the-bit RSS.

**Fig 2:**
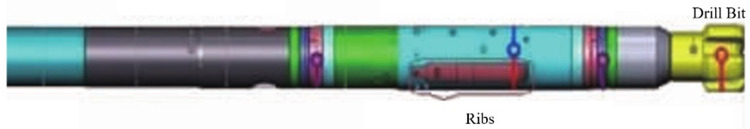
Local mechanism diagram of Welleader system.

The Rotary Steerable System (RSS) enables the drilling tool to follow a pre-designed hole trajectory during downhole drilling [1, 2], which can effectively improve drilling speed, drilling safety and control accuracy [3, 4]. It is a key technology for efficiently completing directional and horizontal wells, and improving the exploration and development efficiency of conventional and unconventional oil and gas reservoirs [5, 6]. At present, the Welleader static push-the-bit RSS developed by China Oilfield Service Co., Ltd. has completed dozens of directional wells and horizontal wells in the Bohai Sea area [7–9]. However, researchers have found that during drilling with the Welleader system, the actual drilling pressure applied at the drill bit is often less than the ’nominal pressure. One important reason for this phenomenon is the rib working resistance of the push-the-bit rotary steerable drilling system. As the RSS inevitably encounters spiral wellbore and creates numerous steps on the well wall (as shown in Fig 1) during operation [10–12]. When the RSS system guides its ribs out (as shown in Fig 2), after the front chamfer surface contacts the wall step, a considerable part of the drilling pressure will be consumed. Continuing to release the hook load will allow the guide ribs to pass the wall step, but it will seriously affect the structure and strength of the downhole tool itself. In addition, if the design of the front chamfer is not reasonable, no matter how much hook weight is released before the rock breaks up at the step, it will not be able to pass the guide ribs through the wall step [13, 14].

This phenomenon is called backing pressure, it can lead to the following problems:

1. Only part of the drilling pressure is applied to the drill bit, resulting in low mechanical drilling speed, or even unable to drill;
2. When the backing pressure phenomenon is serious, the drilling tool will be stationary for a long time, which is not conducive to removing cuttings, and the differential pressure sticking is easy to occur.
3. After the occurrence of backing pressure, if the drilling pressure continue to increase until it lifted, the drilling pressure and torque on the drill bit will suddenly increase, thus causing fluctuations or changes in the tool surface, and the drill bit and drilling tools are easy to be damaged, affecting the efficiency of directional drilling [15, 16].

To address this problem, Fu Chenglin used the finite element method to establish a mechanical model of the contact between the guiding ribs and the well wall for the static push-the-bit RSS, and through orthogonal tests, he determined that the contact pressure between the guiding ribs and the well wall increases with the increase of the elasticity modulus of the well wall and the push-to-recline force, and decreases with the increase of the radius of curvature of the ribs [16]. Shi Yucai considered the two cases of well wall without step and with step, and came to the conclusion that the rotary guiding system has the minimum requirement for nominal drilling pressure, and appropriately increasing the nominal drilling pressure can help to improve the efficiency of drilling pressure transfer, no matter whether there is a step or not in the well wall, if the sum of the guiding ribs’ pushing force is larger, and the larger the friction coefficient of the wall is, the lower the efficiency of the drilling pressure transfer will be. When the well wall has a step, the drilling pressure transfer efficiency is obviously lower than the corresponding value when the well wall does not have a step [13]. By analyzing the force on the lower drilling tool combination, Bo Yubing studied the mechanism of backing pressure generation and found that backing pressure is mainly caused by the frictional resistance between the drilling column and the well wall, and its influencing factors mainly include the borehole trajectory, the degree of cleanliness of the borehole, the combination of drilling tools, the lubricity of the drilling fluid and the type of stratum. It has been verified through field tests that optimization of borehole track and drilling tool combinations, improvement of borehole cleanliness and lubricity of drilling fluids, and adoption of improved downhole tools can effectively reduce the frequency and degree of backing pressure [17]. Yi Guangzhong analyzed four basic ways to mitigate the buttressing effect of the drill column and concluded that mechanical excitation is the best way [18].

At present, in many well sites,, drillers can only choose drilling pressure based on experience, resulting in drilling pressure at the drill bit often not meeting design requirements, seriously affecting drilling efficiency [19–21]. This article optimizes the structure of the front chamfer area and uses a combination of numerical simulation and experimental verification to study the relationship between the forward resistance of the Welleader system when crossing the well wall steps and the front chamfer structure and angle, providing support for the optimization of the forward resistance of the Welleader system.

## Finite element analysis

### Working resistance analysis

**Fig 3:**
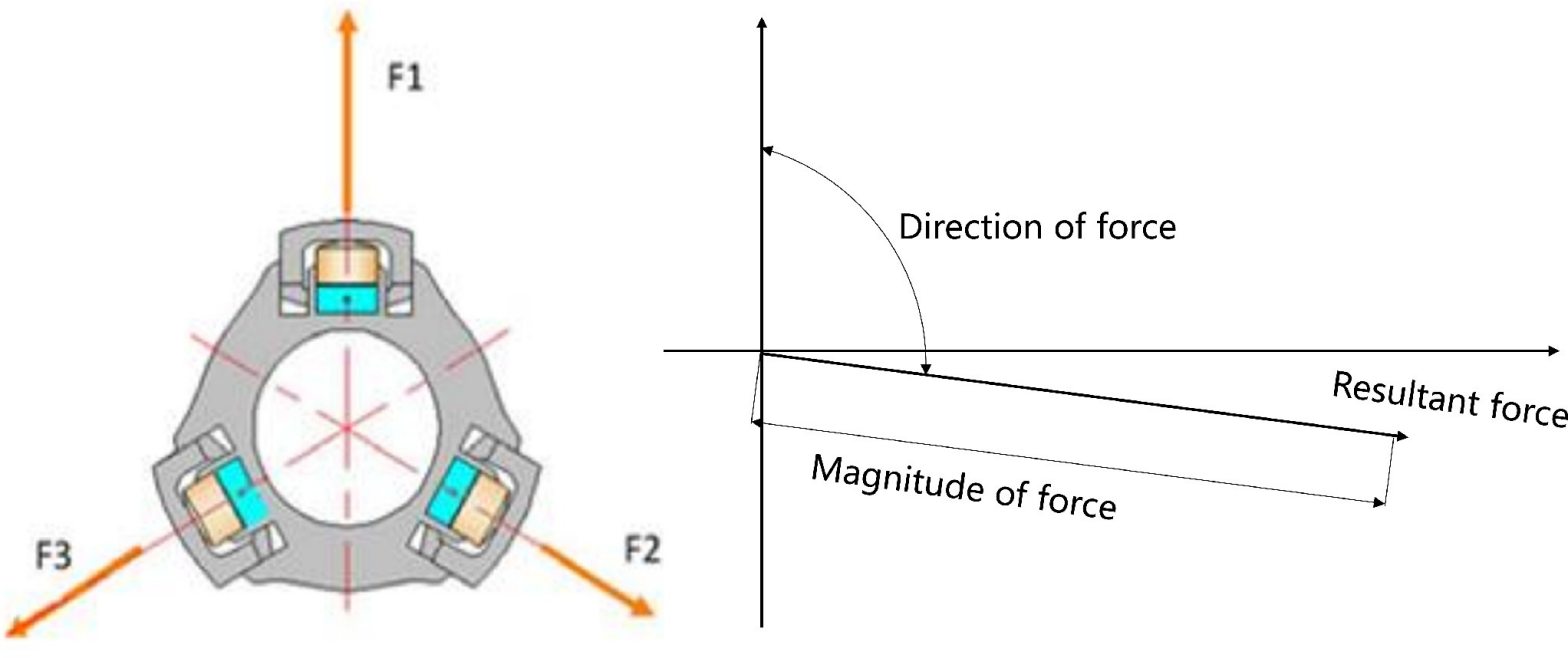
The working principle of the Welleader system.

The working principle of the Welleader system is shown in Fig 3. During its operation, three ribs evenly distributed along the radial direction of the non-rotating outer cylinder are supported on the well wall with different hydraulic pressures. At the same time, the reaction force of the well wall will generate a resultant force on the Welleader system. By controlling the thrust of the three ribs separately, the magnitude and direction of the resultant force can be controlled, thus realizing guided drilling.

**Fig 4:**
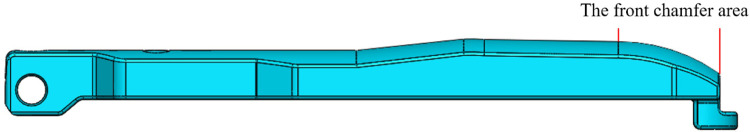
The three-dimensional model of the steering rib.

The three-dimensional model of the steering rib is shown in Fig 4. It adopts a single-plunger articulated structure, the left end is fixed with a pin, and the right end is exerted by a single-plunger to apply thrust. When the ribs working, it will contact the well wall with the left pin hole as the rotation center, and the front chamfer area will first contact the step. Therefore, the front chamfer area is the key part when crossing the step.

**Fig 5:**
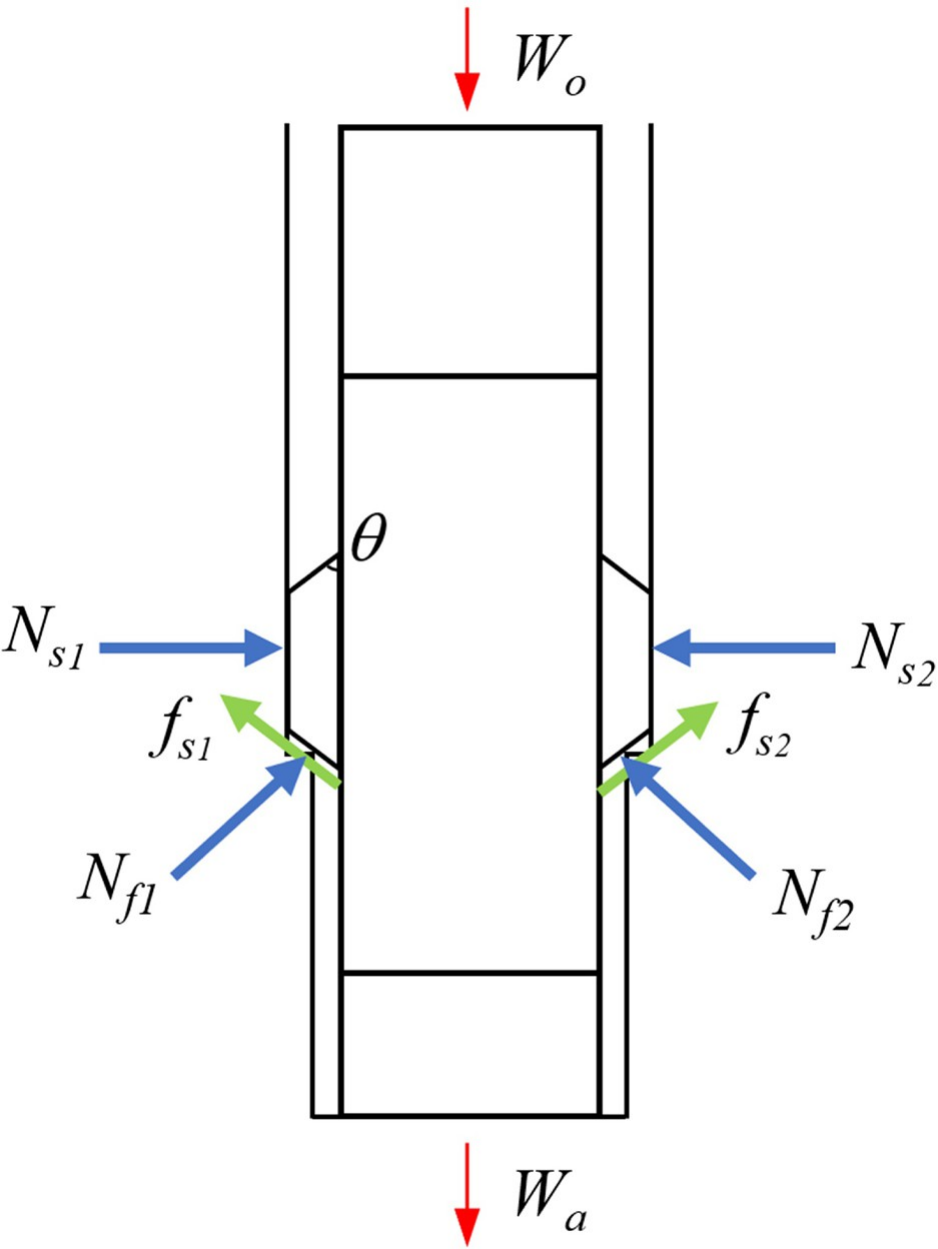
Force analysis of the system.

**Fig 6:**
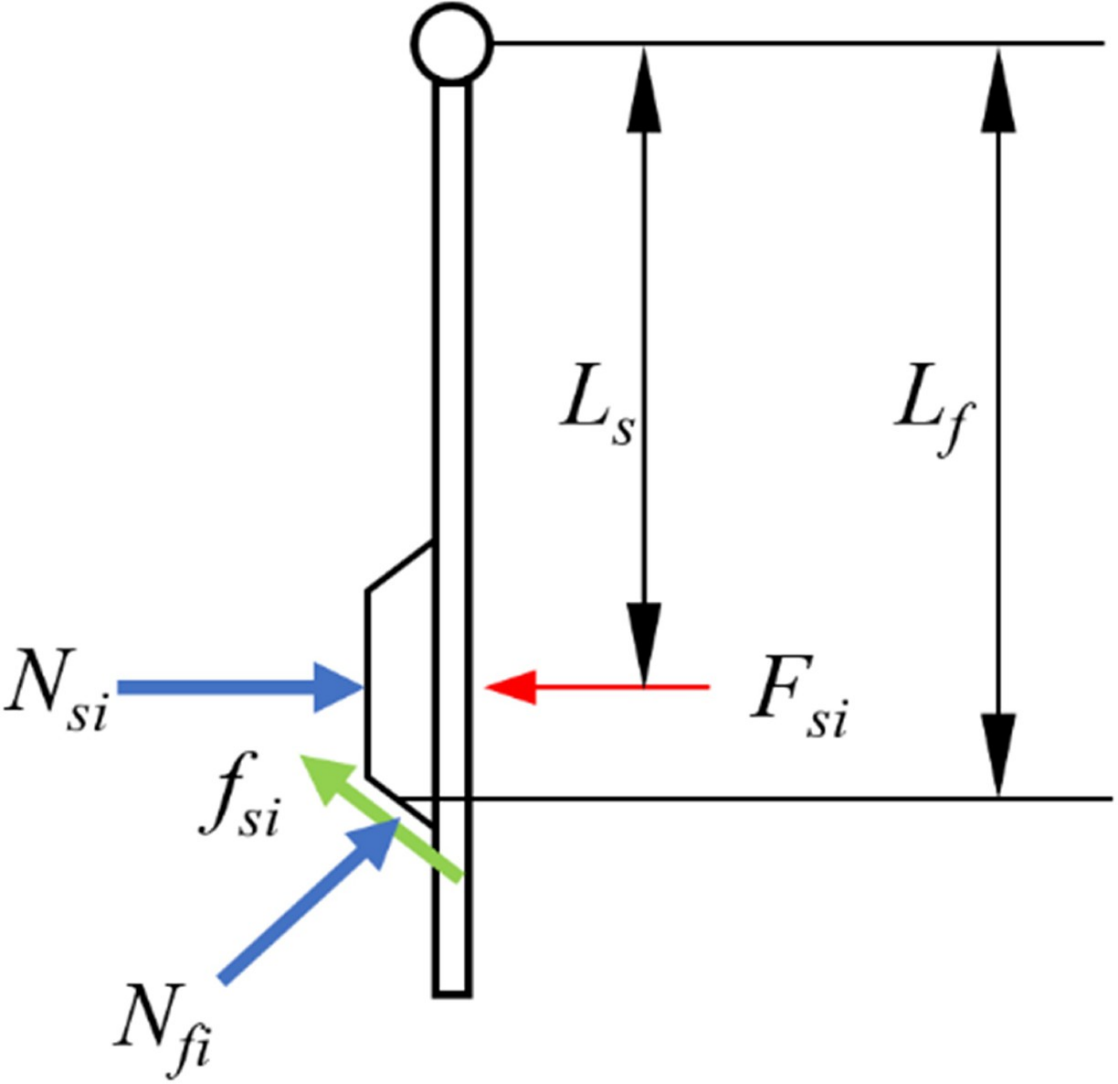
Force analysis of a single rib.

When the front chamfer area contacts the well wall step, the overall force analysis of the system is shown in Fig 5, and the force state of a single rib is shown in Fig 6.

When the ribs start to retract, the working surface will gradually detach from the well wall, and the supporting force Ns on the working surface of the wing ribs will disappear. From the force analysis of the system in Fig 5, it can be seen that all the three ribs need to satisfy the following moment equilibrium equation to rotate around the pin and retract:

$$\left\{\begin{array}{l} \sum_{i=1}^{3}\left(N_{fi}\cos\theta -f_{fi}\sin\;\theta \right)L_{f}=\sum_{i=1}^{3}F_{si}L_{s}\\ f_{fi}=\mu N_{fi} \end{array}\right.$$ (1)

When the front chamfer face passes through the well wall step, all external loads on the system satisfy the following equation:

$$W_{\text{a}}=W_{\text{o}}-\sum_{i=1}^{3}N_{\text{fi}}\sin\theta -\sum_{i=1}^{3}f_{\text{fi}}\cos\theta =W_{\text{o}}-\sum_{i=1}^{3}N_{\text{fi}}(\sin\theta +\mu \cos\theta )$$ (2)

where:

N_f_: Well wall support force applied on the front chamfer face;

f_f_: Well wall friction force exerted on the front chamfer face;

F_s_: Push force exerted on the well wall by the rib;

L_s_: The distance from the center of the rib to the pin;

L_f_: Total length of the rib;

θ: The angle of the front chamfer;

W_a_:Actual drilling pressure, i.e. the drilling pressure acting on the bit;

W_o_: Nominal drilling pressure, i.e. the drilling pressure provided by the drilling rig.

It is finally obtained from Eqs (1) and (2):

$$W_{\text{a}}=W_{\text{o}}-\frac{L_{\text{s}}(\sin\theta +\mu \cos\theta )}{L_{\text{f}}(\cos\theta -\mu \sin\theta )}\sum_{i=1}^{3}F_{\text{s}i}=W_{\text{o}}-\mu_{\text{e}}F_{\text{st}}$$ (3)

where μ_e_ denotes the equivalent friction coefficient, calculated as follows:

$$\mu_{\text{e}}=\frac{L_{\text{s}}(\sin\theta +\mu \cos\theta )}{L_{\text{f}}(\cos\theta -\mu \sin\theta )}$$ (4)

Considering that the actual drilling pressure (Wa) cannot be less than zero, Eq 3 needs to be corrected when the nominal drilling pressure (Wo) is small. The law of variation of the actual pressure with the combined force of the nominal pressure and the thrust force of the guiding ribs is derived, i.e.


$$W_{\text{a}}=\left\{\begin{array}{l} 0\;\left(W_{\text{o}}⩽\mu_{\text{e}}F_{\text{st}}\right)\\ W_{\text{o}}-\mu_{\text{e}}F_{\text{st}}\left(W_{\text{o}}>\mu_{\text{e}}F_{\text{st}}\right) \end{array}\right.$$ (5)

The current Welleader system uses the front chamfer area in a circular arc shape, this shape has the largest value of θ at the initial moment when crossing the well wall steps, with the most serious backing pressure phenomenon. Subsequently, θ gradually decreases, and in the process, it may cause the denominator in Eq 4 to tend to 0+, then the equivalent friction coefficient μ_e_ tends to infinity, and severe backing pressure phenomenon occurs. In addition when the steps are denser, the resistance will show a clear fluctuation trend. Compared with the arc shape, the front chamfer of the polyline is a constant value when crossing the steps of the well wall θ, so the forward resistance in this process is a constant value, and by designing a reasonable front chamfer, the backing pressure phenomenon can be effectively reduced. In addition, even when the steps are more dense, the resistance will not fluctuate. Therefore, the original arc-shaped front chamfer is designed as a folded front chamfer.

### Geometric model and material properties

**Fig 7:**
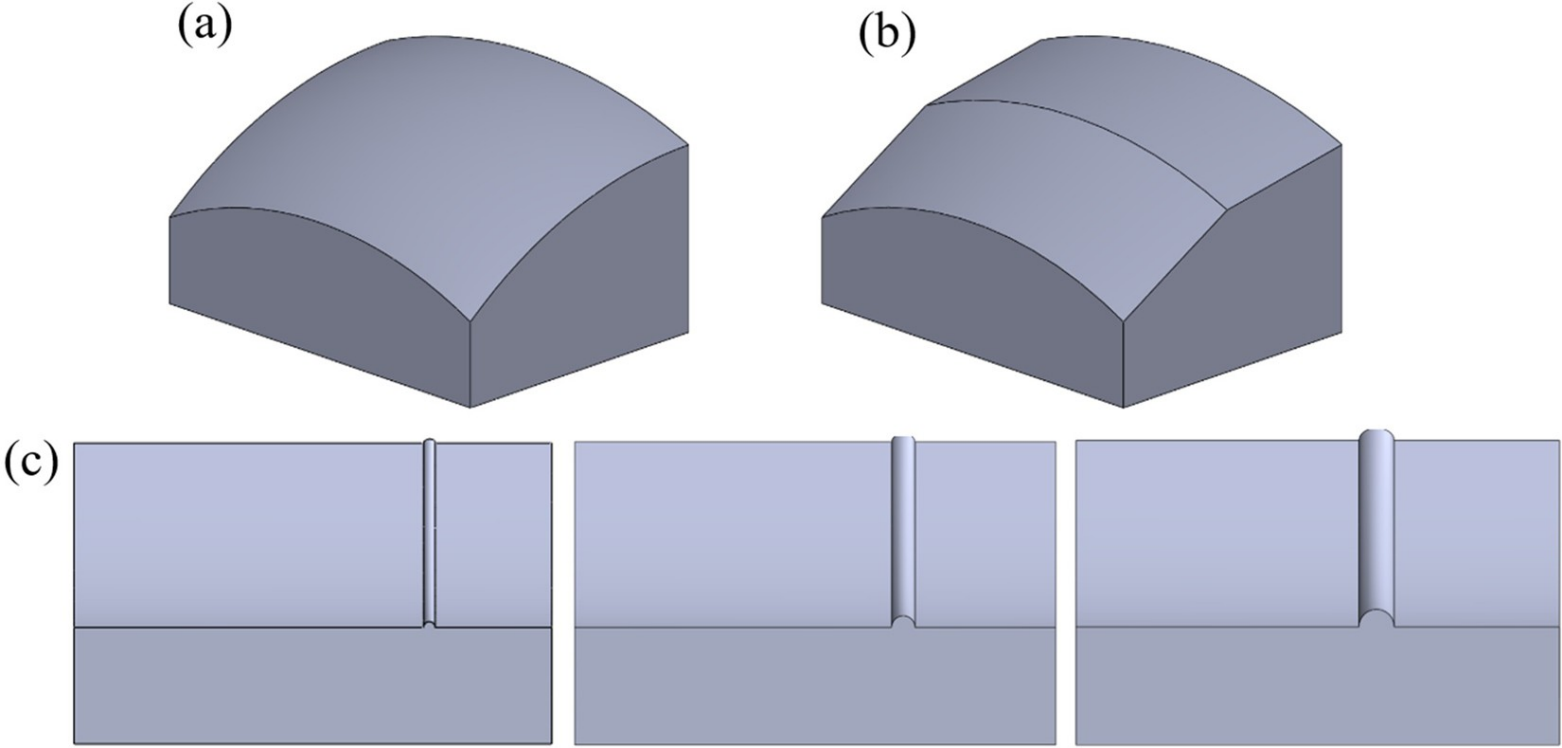
The three-dimensional model of the **(a)** circular arc front chamfer **(b)** polyline front chamfer **(c)** three well wall step model.

The front chamfer area of the Welleader system steering rib was intercepted, and the front chamfer model of the circular arc was reconstructed as the control group. In order to determine the specific angle of the polyline, the front chamfer area is treated with a line within the boundary arc. The finite element software ABAQUS is used to simulate the stress of the model crossing the step at various polyline angle. Due to the correlation between the height of step and the type of drill bit, the drilling technology, the formation being drilled and considering the difference between the diameter of the drill bit and the outer diameter of the RSS, three well wall step models with 1mm, 2mm, and 3mm were established, as shown in Fig 7.

**Fig 8:**
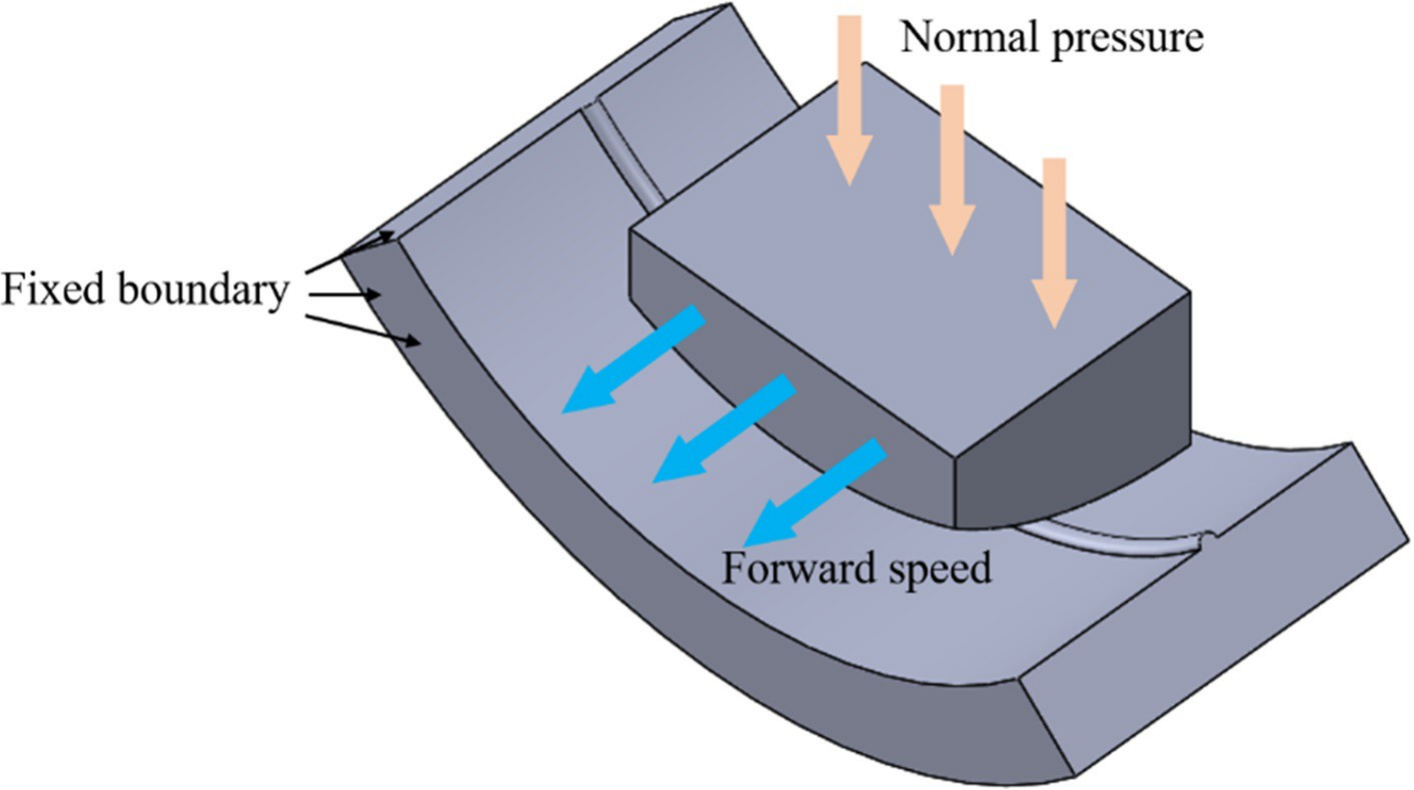
Boundary condition.

The test model and boundary conditions are detailed in Fig 8, and completely fixed constraints are applied to the outer surface of the well wall step model. Normal pressure is applied to the front chamfer model in the Z direction, restricting its three rotational degrees of freedom. Additionally, a forward speed in the Y direction is imparted to the model to simulate crossing the step, allowing for observation of the reaction force on the front chamfer model in the Y direction. The front chamfer model is made of 45 steel, while the wellbore model is constructed from granite. Refer to Table 1 for the basic material parameters [22, 23].

**Table 1:** Physical parameters for finite element analysis.

Model	Material	Dendity(kg·m^-3^)	Elastic modulus(Gpa)	Poisson’s ratio
The front chamfer	45 steel	7890	209	0.269
The well wall step	Granite	2650	40	0.25

#### Simulation results and discussions

**Fig 9:**
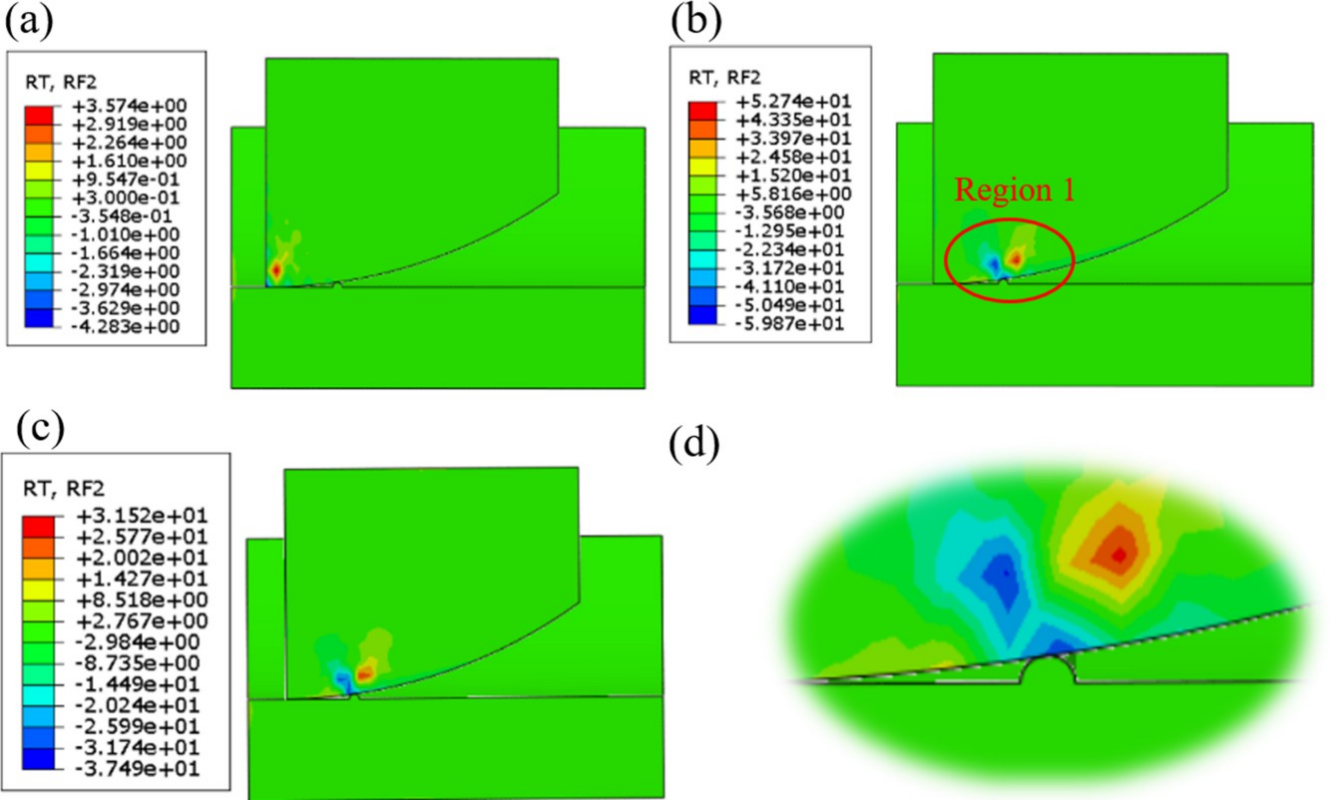
The moment **(a)** Before touching the step **(b)** Touching the step **(c)** After touching the step **(d)** The enlarged diagram of Region 1.

Fig 9 illustrates the force diagram of the arc front chamfer model intersecting the 1mm step, while Fig 9D provides an enlarged view of region 1 in Fig 8B. In the diagram, the blue section denotes the reaction force acting on the model, with the positive direction along the Y-axis. Analysis of Fig 9B reveals that the reaction force is predominantly concentrated within a 5mm radius of the contact point, reaching its peak when the front chamfer model and step make initial contact.

**Fig 10:**
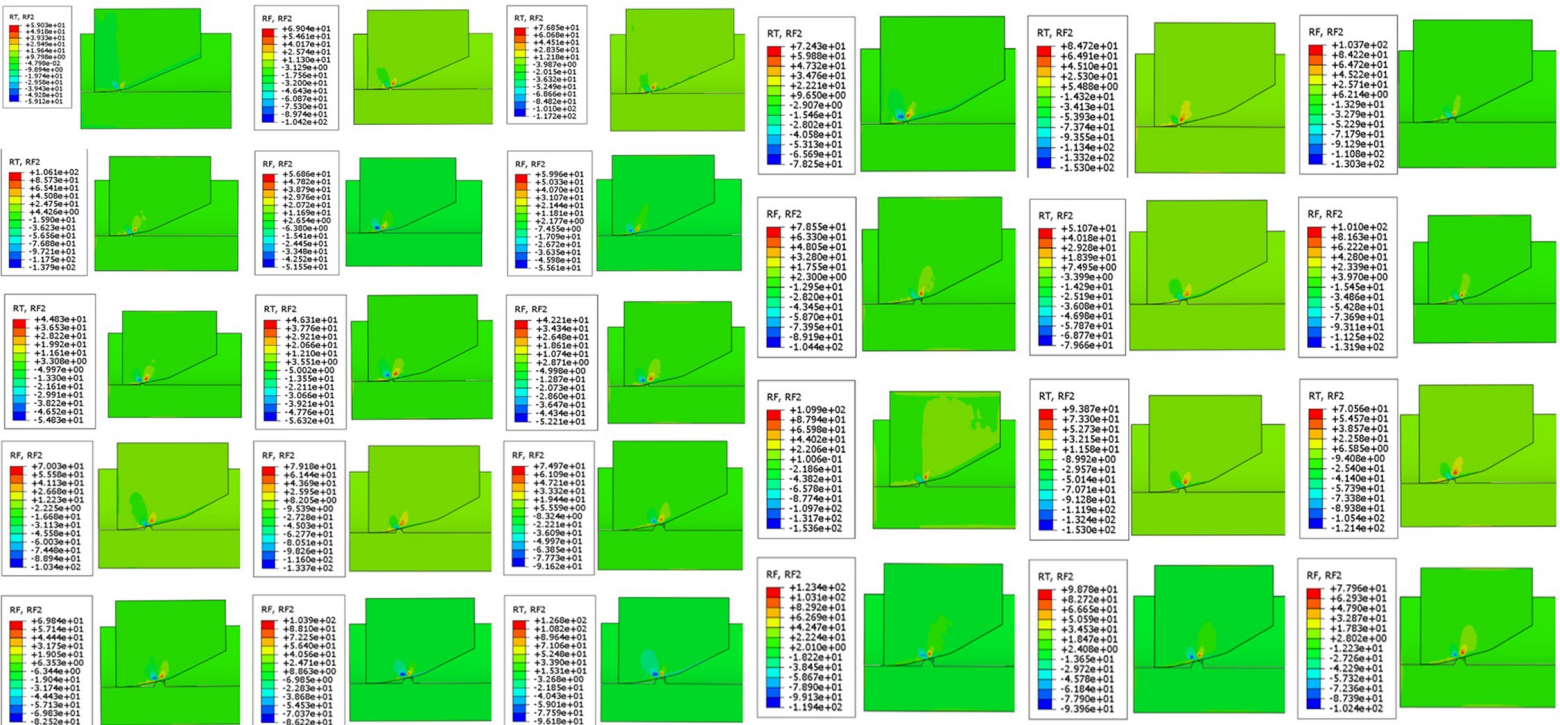
Stress nephogram of different models.

**Fig 11:**
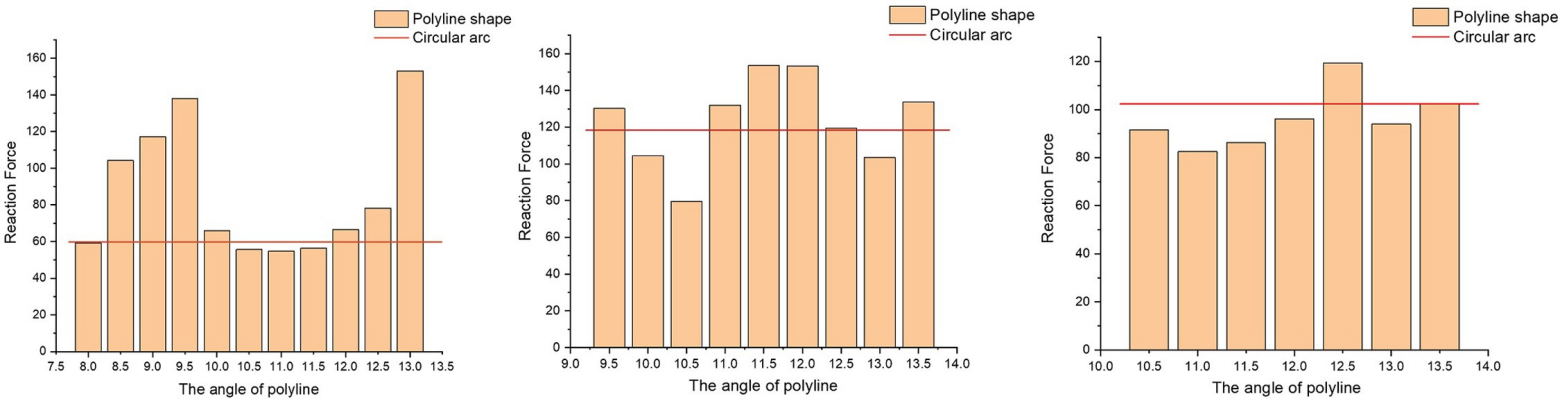
Reaction force of different models cross **(a)** 1mm **(b)** 2mm **(c)** 3mm steps.

Subsequently, 27 sets of test models were created by changing the height of the steps and the angle of the line. The load conditions were maintained constant for simulation. In Fig 10, the stress cloud map of each model group is displayed at the initial contact moment with the step. The reaction force value was extracted and plotted in Fig 11. The red line in Fig 11 is used as a reference to indicate the reaction force of the circular arc front chamfer.

It can be seen that the range of horizontal coordinate in Fig 11(A)–11(C) is different. This is because as the height of the step increases, the minimum angle of the polyline also needs to increase to ensure that the model can smoothly cross the step, so the horizontal coordinate range changes. As can be seen from the Fig 11, when the polyline front chamfer model crosses the step with a height of 1-3mm, the optimal polyline angle is concentrated in the range of 10°-11°. The 10.5° has a good performance in the face of three kinds of height steps. The forward resistance was reduced by 7.1%, 32.8% and 10.4% respectively. This angle basically conforms to the optimal angle in the trend chart in the literature [13], so 10.5° is selected to polyline the front chamfer and process the corresponding test block.

## Experimental procedure

### Experimental material and equipment

**Fig 12:**
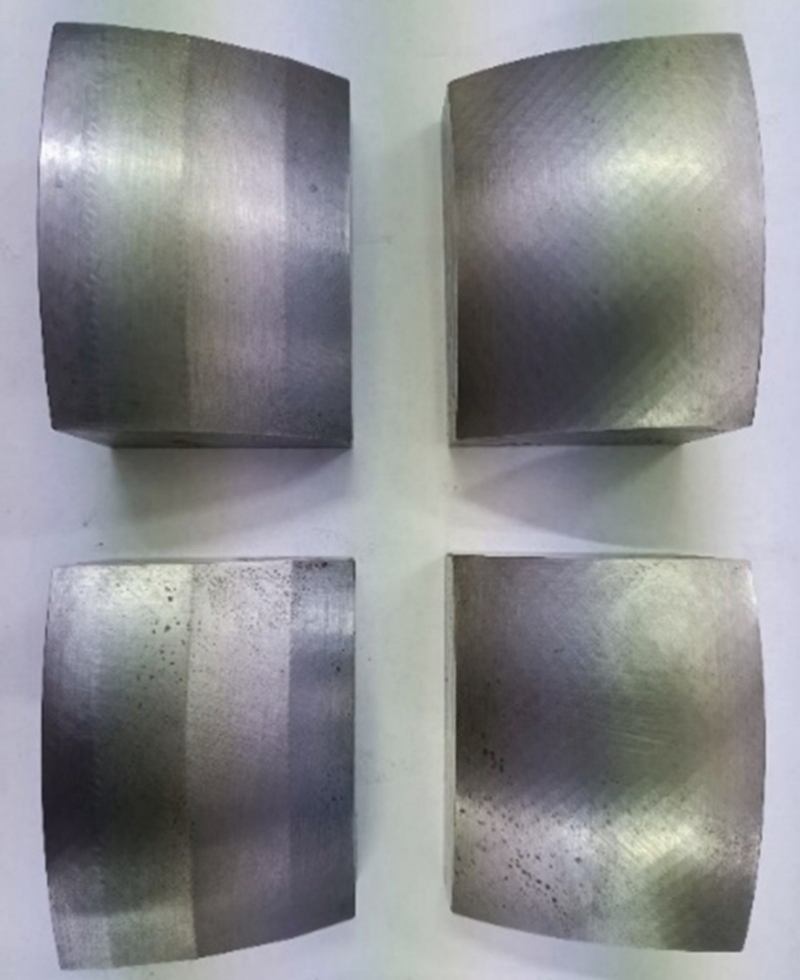
Test block.

The test block was machined with the simulation result. 45 steel was selected as the material and processed using a CNC milling machine. At the same time, a set of test blocks for circular arc were processed as a control group. The test block is shown in Fig 12.

**Fig 13:**
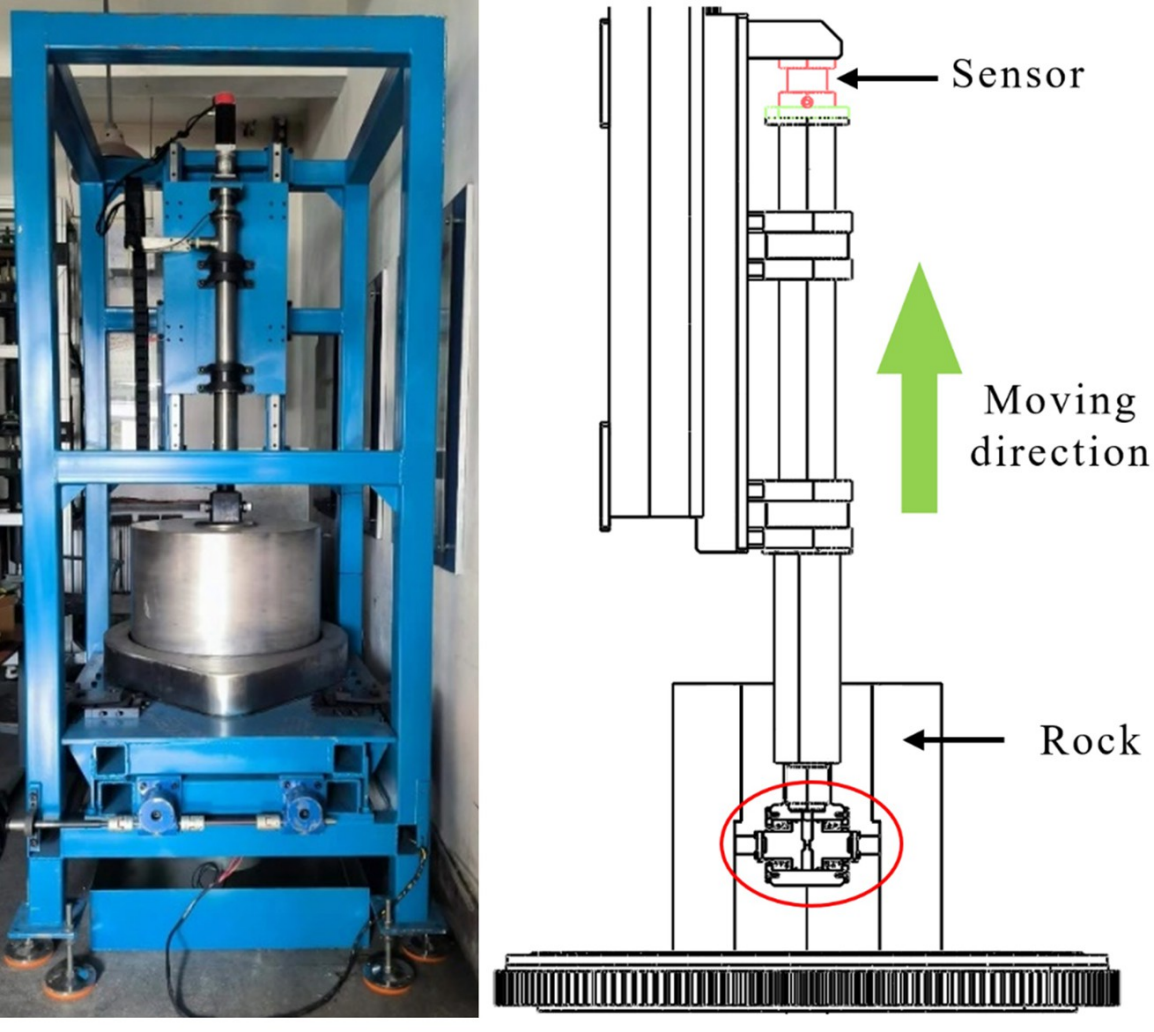
**a** Test apparatus. **b** Schematic diagram of test.

The test was carried out on a self-designed and processed test apparatus. Test apparatus length 2200mm, width 1200mm, height 2600mm. The actual test apparatus and test principle are shown in Fig 13. Before the test starts, the test block is installed on both sides of the test joint, as shown in Fig 16, and then extended into the central hole of the rock below. High-pressure gas is injected through an external air compressor to push the test block out and into contact with the rock, and then the motor is started to make the test block rise and fall in the hole. The real-time resistance change is detected through the upper pull pressure sensor. After the test is completed, replace the test block type and repeat the above steps. In order to reduce the test error, the average value of each group of test measurements was taken as the result after 3 times.

**Fig 14:**
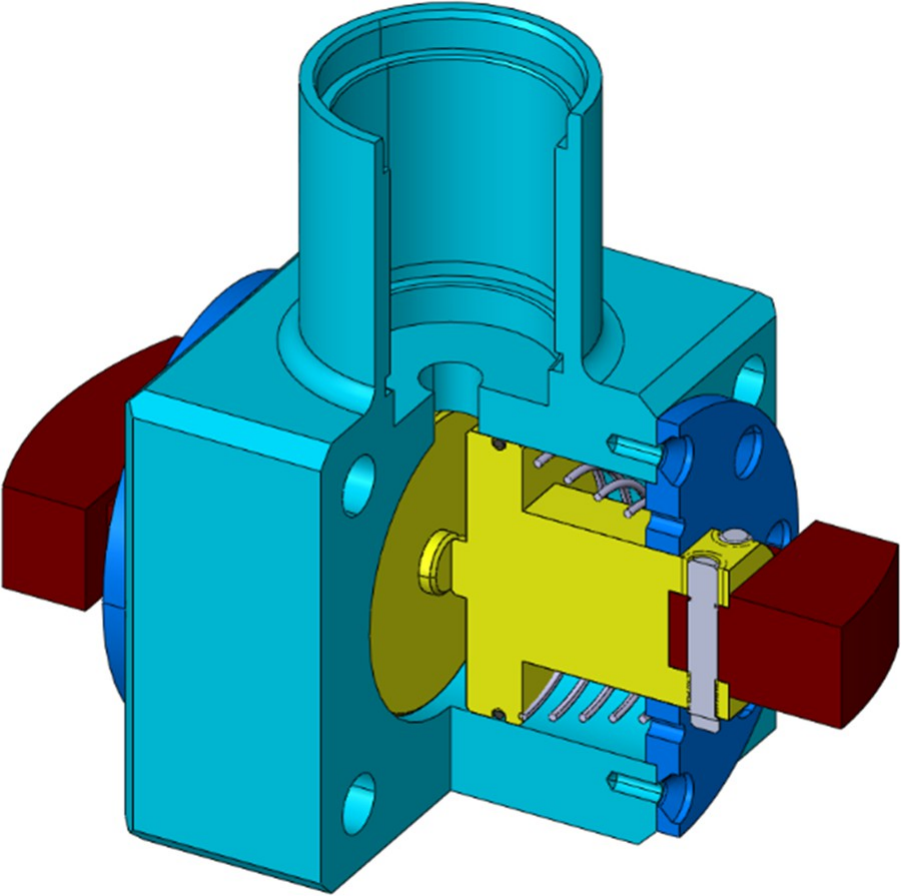
Test joint.

**Fig 15:**
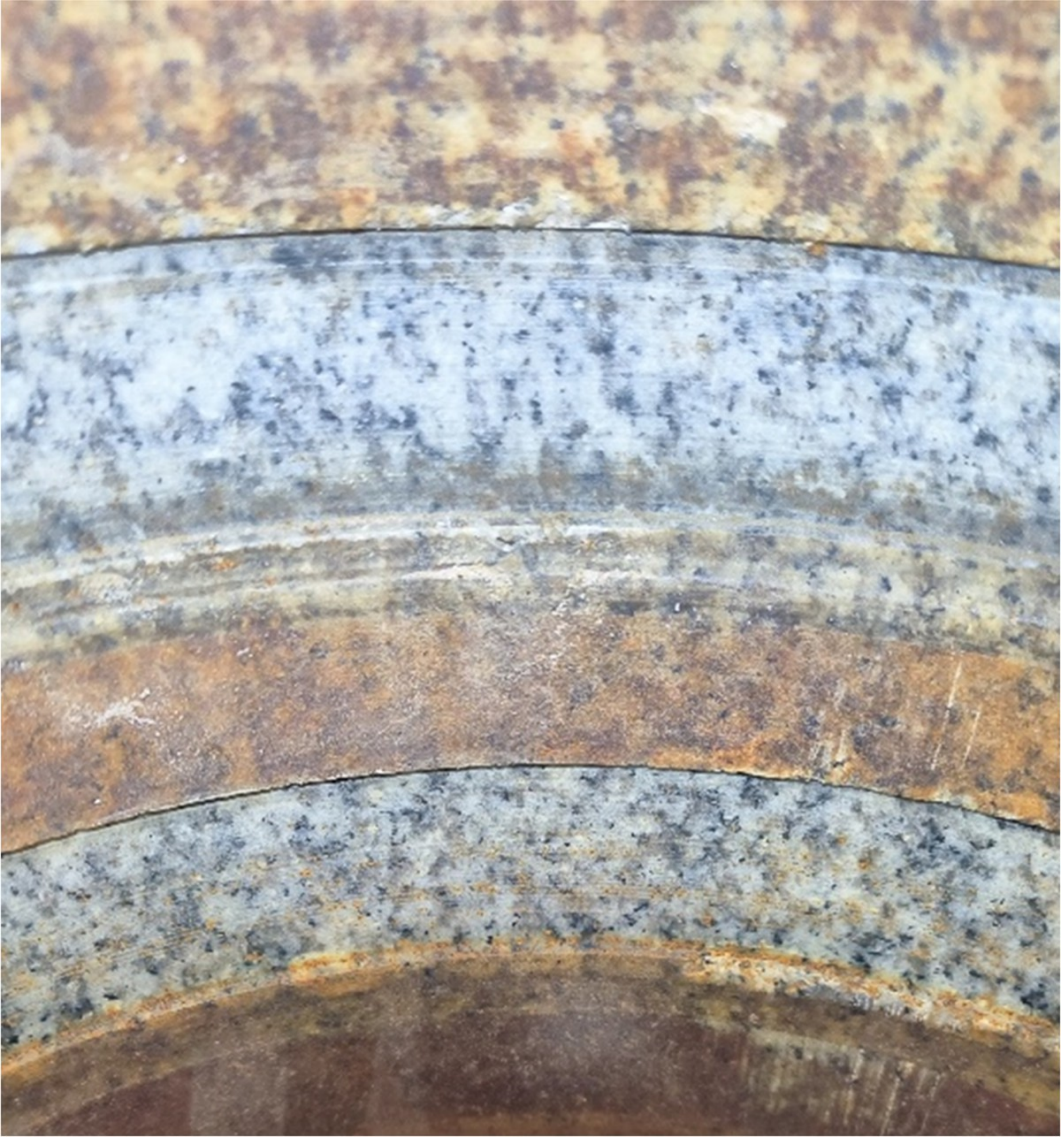
The well wall step.

**Fig 16:**
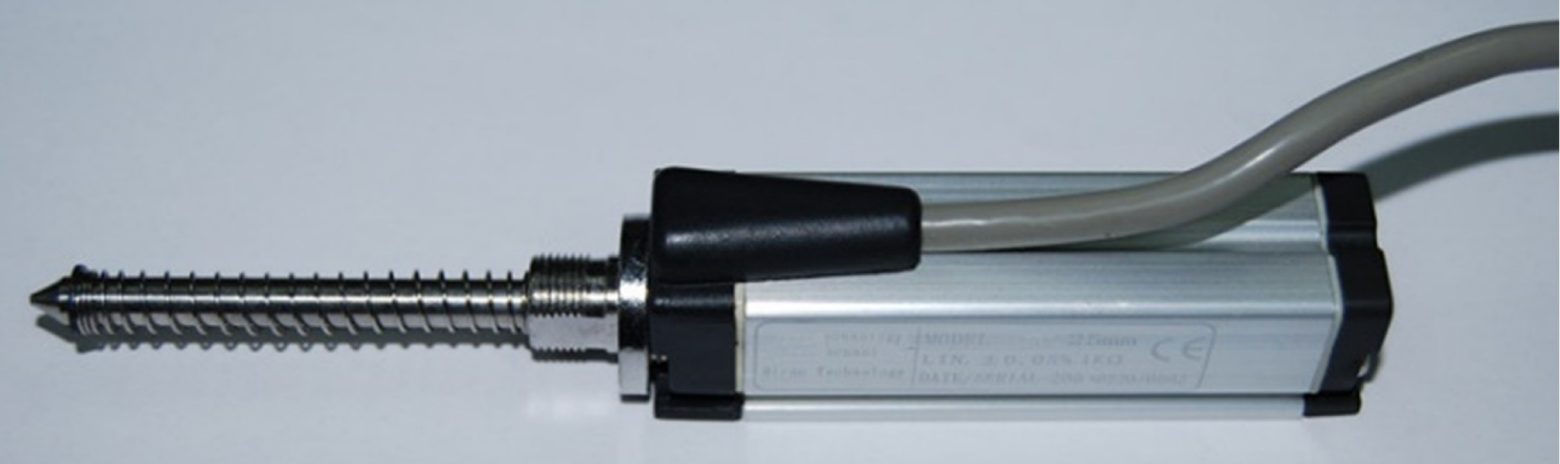
The self-resetting displacement sensor.

Fig 14 shows the test joint which is the enlarged diagram of region 2 in Fig 13. The rock used in the test is granite with a through hole in the center. Steps of different heights are arranged on the surface of the hole wall, as shown in Fig 15. The height of the steps is measured by the self-resetting displacement sensor shown in Fig 16, and the specific values are shown in Table 2.

**Table 2:** Table of step height.

Step number	Step height
1	3.47mm
2	1.01mm
3	2.09mm

## Experimental results and discussion

**Fig 17:**
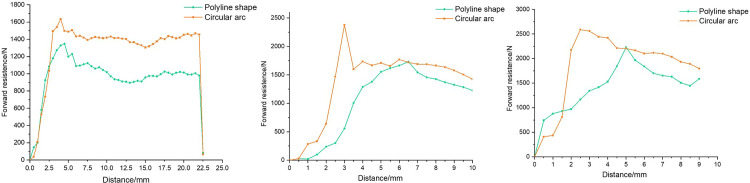
Reaction force of two test blocks cross **(a)** 1mm **(b)** 2mm **(c)** 3mm steps.

Fig 17 shows the relationship between forward resistance and distance under the condition of two test blocks crossing 1-3mm steps. The orange curve represents the circular arc test block, and the green curve represents the polyline test block. The initial value has been subtracted from the weight of the test part, resulting in a value of 0.

The overall trend of the curve shows a rapid increase from 0 to the highest point, followed by a decrease. This is due to the fact that at the beginning of the test the block changes from rest to motion, which causes the resistance to advancement to increase rapidly from zero. As the distance traveled increases, the block touches a step in the well wall, at which point the resistance to advancement peaks and then decreases. This trend is consistent with that obtained from the simulation.

In addition, in the three cases, the peak time of the polyline test block is later than that of the circular arc test block, which accords with the design principle.

The mean value and peak value of the forward resistance when the two tests were about to cross different steps were extracted respectively, as shown in Table 3. It can be seen that under the 1-3mm step, the forward resistance peak value of the polyline test block is decreased by 19.8%, 25.0% and 13.9%, respectively, and the mean value is decreased by 17.4%, 27.2% and 24.1%, respectively. Among these, the decrease in resistance is most noticeable when crossing the 2mm step, which is similar to the simulation results.

**Table 3:** Comparison of forward resistance.

		Circular arc/N	Polyline/N	Comparison
1mm	Peak value	1632.4	1347.6	19.8% reduction
	Mean value	1416.7	980.3	30.8% reduction
2mm	Peak value	2378.1	1731.6	25.0% reduction
	Mean value	1341.5	1005.8	27.2% reduction
3mm	Peak value	2587.1	2228.0	13.9% reduction
	Mean value	1809.9	1374.6	24.1% reduction

## Conclusion

1. Through theoretical analysis, the value of θ changes when the circular arc shaped front chamfer crosses the step of the well wall, which may lead to the equivalent friction coefficient μe tends to infinity, and serious backing pressure phenomenon occurs. On the other hand, the θ value of the polyline front chamfer is constant when crossing the step of the well wall, so the forward resistance is constant in this process, and the pressure support phenomenon can be effectively reduced by designing a reasonable front chamfer angle.
2. When the polyline front chamfer model crosses the step with a height of 1-3mm, the optimal polyline angle is concentrated in the range of 10°-11°. The 10.5° has a good performance in the face of three kinds of height steps.
3. Through experiments, it was found that the forward resistance peak value of the polyline test block is decreased by 19.8%, 25.0% and 13.9%, respectively, and the mean value is decreased by 30.8%, 27.2% and 24.1%, respectively.

Among these, the decrease in resistance is most noticeable when crossing the 2mm step, which is similar to the simulation results.

## Supporting information

S1 Table Statistics of simulation results of different models reaction force. (XLSX)

S2 Table Statistics of test results of different models reaction force. (XLSX)
